# Predicting Range Shifts of Five *Alnus* (Betulaceae) Species in China Under Future Climate Scenarios

**DOI:** 10.3390/plants14111597

**Published:** 2025-05-24

**Authors:** Wenjie Yang, Zhilong Huang, Chenlong Fu, Zhuang Zhao, Xiaoyue Yang, Quanjun Hu, Zefu Wang

**Affiliations:** 1 State Key Laboratory of Tree Genetics and Breeding, Co-Innovation Center for Sustainable Forestry in Southern China, College of Ecology and Environment, Nanjing Forestry University, Nanjing 210037, China; ywj.chuck@gmail.com (W.Y.); huang20010224@gmail.com (Z.H.); njzhaozhuang@njfu.edu.cn (Z.Z.); yangxiaoyue@njfu.edu.cn (X.Y.); 2 Key Laboratory of Bio-Resource and Eco-Environment of Ministry of Education, College of Life Sciences, Sichuan University, Chengdu 610065, China

**Keywords:** climate change, *Alnus*, species distribution models, suitable habitats

## Abstract

Climate change poses significant challenges to forest biodiversity by altering species distributions. This study employed the MaxEnt model to predict the current and potential future suitable habitats of five *Alnus* species in China under four Shared Socioeconomic Pathways. Model accuracy was high, with temperature seasonality identified as the most influential variable. In addition, predicted range shifts showed species-specific patterns, with most species expanding toward higher latitudes and elevations. In contrast, *Alnus ferdinandi-coburgii* exhibited consistent habitat contraction. These findings enhance understanding of the climatic responses of *Alnus* species and provide a scientific basis for targeted conservation and management strategies under future climate change, and may offer insights into habitat responses of *Alnus* species in other temperate regions.

## 1. Introduction

Global climate change significantly threatens biodiversity by altering the habitats and distribution patterns of species, resulting in cascading impacts on ecosystem functions and human well-being [1,2,3]. Rising temperatures, shifting precipitation regimes, and extreme weather events have been documented to drive species toward higher latitudes or elevations, disrupting existing ecological networks [4,5,6]. For temperate and boreal forest species, these changes may exacerbate habitat fragmentation and reduce adaptive capacity, particularly for genera with narrow ecological niches [6,7,8]. Understanding how climate change impacts species distributions is critical for developing conservation strategies that mitigate biodiversity loss [9].

Species distribution models (SDMs) have emerged as essential tools for predicting potential habitat suitability under current and future climatic conditions [10,11,12]. Among these, the Maximum Entropy (MaxEnt) model has gained prominence due to its robustness in handling limited occurrence data and its ability to identify key environmental drivers of species distributions [5,13,14]. MaxEnt has been successfully applied to forecast range shifts in diverse plant taxa, including keystone forest trees, under various climate scenarios [15,16].

The genus *Alnus* (Betulaceae), commonly known as alders, comprises ecologically and economically vital species distributed across the Northern Hemisphere, including East Asia, Europe, and North America. This widespread distribution highlights the ecological importance of *Alnus* on a global scale and underscores the relevance of regional studies to broader conservation contexts. Alders are renowned for their nitrogen-fixing symbiosis with *Frankia* bacteria, enhancing soil fertility and facilitating ecosystem restoration in degraded landscapes [17,18]. Species such as *Alnus cremastogyne* and *Alnus hirsuta* are critical for riparian zone stabilization, while others like *Alnus trabeculosa* provide high-quality timber for industrial applications [19,20,21]. Despite their ecological significance, comprehensive studies on the potential distribution of *Alnus* species under climate change remain limited. Previous research has focused predominantly on single species or regional scales, leaving gaps in understanding the genus-wide responses to global change [13,22,23].

This study addresses these gaps by modeling the current and future suitable habitats of five *Alnus* species (*Alnus cremastogyne* Burkill, *A*. *ferdinandi-coburgii* C. K. Schneid., *A*. *mandshurica* (Callier) Hand.-Mazz., *A*. *trabeculosa* Hand.-Mazz., and *A*. *hirsuta* Turcz. ex Rupr.) across China. Using the MaxEnt framework, we aim to (1) identify key climatic variables shaping their distributions, (2) predict potential range shifts under multiple future climate scenarios, and (3) assess conservation priorities for these species. Our findings will provide actionable insights for preserving *Alnus* genetic resources and guiding sustainable forest management in the face of climate uncertainty.

## 2. Results

### 2.1. Model Accuracy and Key Environmental Variables

**Figure 1:**
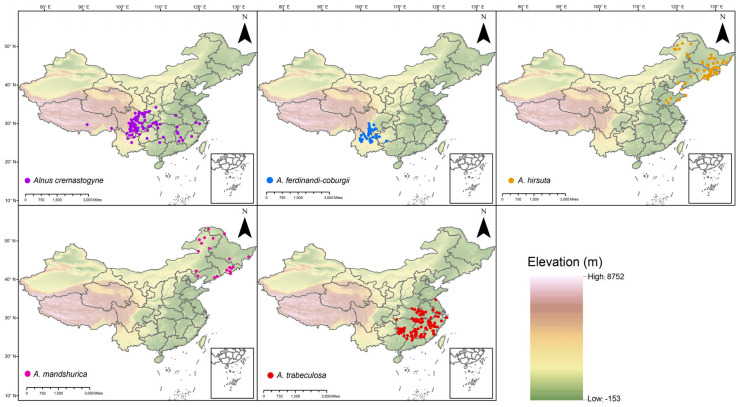
Distribution map of five *Alnus* species in China. Dots indicate the distribution of species.

In this study, we selected five representative *Alnus* species (*A. cremastogyne*, *A. ferdinandi-coburgii*, *A. mandshurica*, *A. trabeculosa*, and *A. hirsuta*) in China and obtained their occurrence records from public databases for modeling (Figure 1). These five species were selected because they represent the major ecological and geographic lineages of *Alnus* in China, and they have sufficient occurrence records to support reliable modeling. Modeling the current distribution of *Alnus* species in China showed high predictive performance, with AUC and TSS values greater than 0.91 for all species (Table 1), indicating that the MaxEnt model was highly effective in predicting their potential distribution areas. After screening, four main environmental factors were selected for each species for use in model construction (Table 2). Among these, temperature seasonality (bio04) emerged as the common and most crucial environmental variable for all *Alnus* species, with an average contribution of 32.84%, making it the most impactful variable (Table 2). This suggests that bio04 encapsulates critical climatic variability that is not captured by the other variables. Annual precipitation (bio12) influenced the potential distribution of three species. Notably, the precipitation of the driest quarter (bio17) was the primary contributing factor for *A. trabeculosa*, with a contribution of 89% in its model (Table 2).

**Figure 2:**
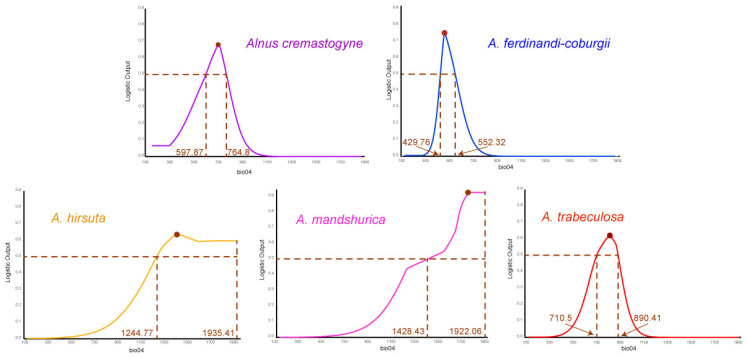
Response curves of temperature seasonality (bio04) affecting the distribution of five *Alnus* species. The range of logistics greater than 0.5 has been shown in figure.

By examining the environmental variables selected for the five species and their corresponding thresholds associated with the highest suitability probabilities, temperature and precipitation variables were found to exert a relatively balanced influence on their distributions (Table 2). However, minor interspecific differences in precipitation requirements were observed among species. Generally, most *Alnus* species prefer cooler and drier habitats, indicating that both temperature and precipitation variables are essential determinants of their distribution. Among these environmental variables, temperature seasonality (bio04) was identified as the most influential factor affecting all five species (Table 2). The response curves illustrating the influence of bio04 on species distribution are presented in Figure 2. We found that optimal suitability for *A. hirsuta* occurred within the highest bio04 range among the five species, specifically 1244.77–1935.41 (Figure 2). *A. mandshurica* was most suitable for distribution within a bio04 range of 1428.43–1922.06. In contrast, *A. cremastogyne*, *A. ferdinandi-coburgii*, and *A. trabeculosa* exhibited relatively narrower optimal bio04 suitability ranges, specifically 597.87–764.80, 429.76–552.32, and 710.50–890.41, respectively (Figure 2). These differences suggest that the five *Alnus* species may exhibit divergent responses to environmental variables due to their distinct geographical distributions.

### 2.2. Predicted Potential Suitable Distribution of the Alnus Species

**Figure 3:**
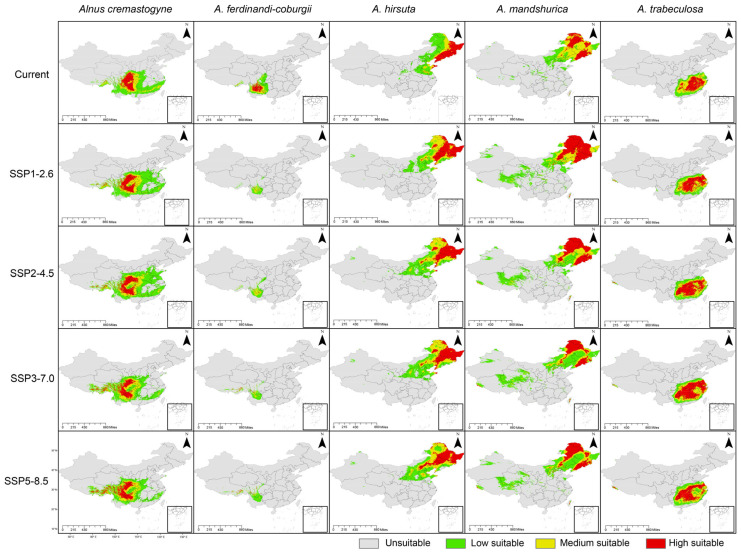
Potential suitable distributions map of five *Alnus* species under the current climate and four future climate scenarios (SSP1-2.6, SSP2-4.5, SSP3-7.0, and SSP5-8.5). Suitability levels are represented by different colors: gray for unsuitable ranges, green for low suitability, yellow for medium suitability, and red for high suitability.

The potential suitable distribution areas of *Alnus* species under both current and future climatic scenarios are illustrated in Figure 3, with habitat suitability categorized into four levels: high, medium, low, and non-suitable zones. Under the current climate conditions, there is a clear geographical separation in the distribution ranges of the five species. *A. hirsuta* and *A. mandshurica* occupy northeastern high-latitude regions, while *A. cremastogyne* and *A. ferdinandi-coburgii* are restricted to southwestern low-latitude regions. Moreover, *A. trabeculosa* shows a distinct adaptation to the southeastern Yangtze River Basin (Figure 3). Among these species, *A. mandshurica* has the largest total suitable habitat area and the widest high-suitability zone, covering 209.37 × 10^4^ km^2^ and 54.93 × 10^4^ km^2^, respectively (Table 3). Meanwhile, *A. ferdinandi-coburgii* exhibits the smallest predicted suitable area, encompassing only 56.10 × 10^4^ km^2^ (Table 3). The predicted responses of the five *Alnus* species to future climate scenarios varied markedly, with no single climate scenario consistently favoring the range expansion of all species (Figure 3, Table 3). This suggests species-specific climate preferences rather than a uniform trend in distribution changes. Notably, we found that the suitable distribution area of *A. ferdinandi-coburgii* under all future climate scenarios was consistently smaller than its current distribution (Table 3).

### 2.3. The Expansion and Contraction of the Distribution Area of Alnus Species

**Figure 4:**
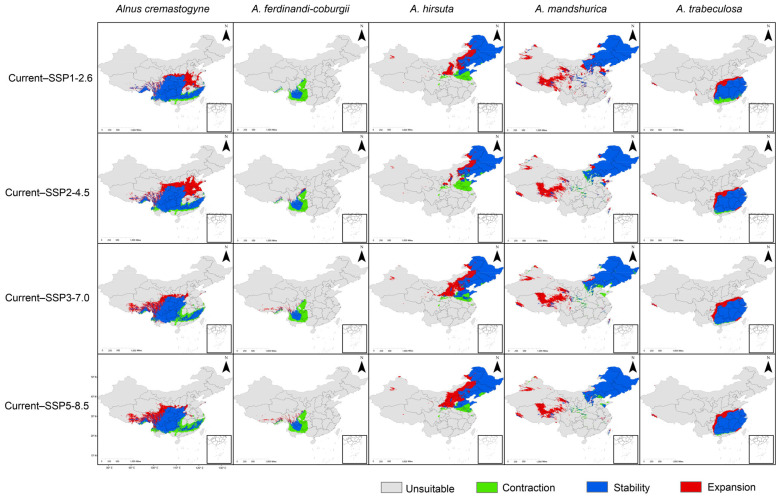
Distribution changes of five *Alnus* species from the current climate to four future climate scenarios (SSP1-2.6, SSP2-4.5, SSP3-7.0, and SSP5-8.5). Gray represents unsuitable ranges, green indicates contraction ranges, blue represents stable ranges, and red indicates expansion ranges.

To further investigate the suitable distribution areas of *Alnus* species, we analyzed the expansion and contraction of potential distribution ranges for different species under various future climate scenarios (Figure 4, Table 4). Under the scenario of climate warming, *Alnus* species are predicted to shift toward higher elevations and latitudes, and a substantial loss of suitable distribution areas is expected in the southern part of the distribution range of *A. hirsuta* and the eastern part of the distribution range of *A. ferdinandi-coburgii* (Figure 4).

For most species, the area of range expansion was greater than that of contraction. Specifically, *A. cremastogyne* demonstrated significant potential range expansion under SSP2-4.5, gaining 84.71 × 10^4^ km^2^ of potentially suitable habitat. More modest potential expansions occurred under SSP1-2.6, SSP3-7.0, and SSP5-8.5, suggesting optimal adaptability to moderate emission scenarios. A similar potential response pattern was observed in *A. mandshurica* (Table 4). Meanwhile, *A. trabeculosa* exhibited its maximum potential gain under SSP3-7.0 (27.25 × 10^4^ km^2^) (Table 4), while *A. hirsuta* achieved its largest potential expansion under SSP5-8.5 (71.15 × 10^4^ km^2^) (Table 4). In contrast, *A. ferdinandi-coburgii*, for which the area of expansion is smaller than the area of contraction under all scenarios, showed a general decrease in potential suitability across all future scenarios. The most significant contraction occurred under SSP3-7.0, with a loss of 38.42 × 10^4^ km^2^, indicating sensitivity to moderate climate warming scenarios (Table 4, Figure 4). This divergence highlights distinct ecological strategies in responding to climatic stressors, with regional climate drivers shaping these contrasting trajectories.

## 3. Discussion

Species distribution models (SDMs) have been widely validated and applied for ecological prediction across spatial and temporal scales [24,25]. In this study, we followed modeling strategies and variable selection approaches that have been successfully used in studies of other tree species, such as *Carpinus* [26], *Monotheca* [27], and *Rhizophora* [28]. While these references focus on different species, they share methodological frameworks with our approach. The MaxEnt models constructed for the five *Alnus* species yielded high predictive accuracy, with AUC and TSS values exceeding 0.91 (Table 1). A similar level of performance was reported in a previous study of *A. cremastogyne* [13], which supports the reliability of our models, although that study focused on a single species.

### 3.1. Assessment of MaxEnt Model

Among environmental variables evaluated, temperature seasonality (bio04) emerged as the predominant factor influencing habitat suitability across all studied *Alnus* species, highlighting the pivotal role of temperature fluctuations in determining ecological niches. Temperature variations can directly affect plant physiological processes such as photosynthesis, transpiration, and seed germination, all of which play critical roles in shaping the ecological distribution of these species [29,30,31].

Additionally, annual precipitation (bio12) and precipitation of the driest quarter (bio17) significantly influenced species distributions. This reflects the dual constraint of temperature and water availability on potential habitat suitability. The significance of annual precipitation (bio12) in shaping species distributions has been widely acknowledged across diverse taxa [14,15,32]. For example, *A. mandshurica* and *A. hirsuta*, both distributed in Northeast China, are strongly associated with bio12, which aligns with their dependence on stable water supply in cold-temperate climates (Table 2). In comparison, precipitation during the driest quarter (bio17) captures seasonal water limitation and is particularly relevant for species (*A. cremastogyne* and *A. ferdinandi-coburgii*) occurring in monsoonal or seasonally dry regions [33]. Reduced moisture availability during the driest quarter can severely impact seedling survival, root growth, and drought resistance, especially in low-latitude environments where dry seasons are pronounced [34,35,36,37]. These findings demonstrate that although temperature and water availability are broadly acknowledged as limiting factors, their relative roles differ by species. Our models help to quantify these species-specific patterns and reveal climatic sensitivities that are difficult to capture through observation alone.

### 3.2. Current and Future Potential Distribution Range of the Alnus Species

Under current climatic conditions, the modeled distribution patterns of *Alnus* species in China exhibit clear regional differentiation, reflecting species-specific adaptations to diverse ecological niches. *A. hirsuta* and *A. mandshurica,* predicted to dominate northeastern China, adapted to regions characterized by pronounced seasonal temperature fluctuations. In contrast, *A. cremastogyne* and *A. ferdinandi-coburgii* are adapted to milder conditions in southwestern China, whereas *A. trabeculosa* inhabits the southeastern regions. Projected climate scenarios indicate notable distribution shifts for these species, primarily toward higher altitudes and latitudes, as a result of increasing temperatures and changes in precipitation patterns. Species distributed in Northeast China are associated with habitats exhibiting greater temperature fluctuations, whereas those in Southwest, South, and East China tend to occur in environments with relatively smaller temperature variations. Several *Alnus* species, such as *A. cremastogyne*, *A. mandshurica*, *A. hirsuta*, and *A. trabeculosa*, exhibited notable potential range expansions under various climate scenarios. These potential expansions may be attributed to broader climatic tolerances and ecological flexibility. For instance, *A. mandshurica* and *A. hirsuta* possess strong cold tolerance and are able to survive across a wide range of temperature regimes [38,39], which facilitates their potential expansion toward higher latitudes. Similarly, *A. cremastogyne* and *A. trabeculosa* can tolerate varying moisture conditions [13,19,40], allowing them to adapt to future changes in precipitation and expand their ranges into suitable new habitats. These expansions may be attributed to broader ecological tolerances or more flexible habitat preferences, enabling these species to follow suitable conditions under shifting climates.

Conversely, *A. ferdinandi-coburgii* faces substantial reductions in potentially suitable habitats, underscoring its vulnerability to climatic stressors. This species is primarily distributed at relatively high elevations and exhibits a narrow climatic niche [41], with limited tolerance to temperature and moisture fluctuations. Such specialization constrains its capacity to migrate or persist under rapid climate change. The projected habitat loss for this species is likely attributable to its current distribution at relatively high elevations, which are particularly sensitive to warming-induced ecological changes. Although *A. ferdinandi-coburgii* exhibits a migration trend toward higher altitudes, especially towards the Qinghai-Tibet Plateau (Figure 4), the extreme elevation of this region poses significant constraints on successful migration [42,43,44]. Such large elevation gradients hinder rapid adaptation and successful colonization, thereby limiting the potential for range expansion and leading to overall potential habitat contraction. These predicted distribution shifts align closely with patterns observed in other mountain and forest ecosystems under similar climatic pressures [45,46].

### 3.3. Implications for Conservation and Forest Management

The varied responses of *Alnus* species to climate change highlight the necessity for tailored conservation and management strategies. Anticipated shifts in the distribution of species toward higher altitudes and latitudes indicate adaptive responses that help maintain optimal climatic conditions, a phenomenon widely documented among temperate forest species affected by global warming [1,47,48]. For example, *A. ferdinandi-coburgii*, which is projected to undergo significant habitat contraction in high-elevation regions, should be prioritized for in situ conservation in the remaining suitable areas. Where feasible, measures such as targeted habitat restoration, ex situ preservation, or even assisted migration may be considered to prevent further population decline. In contrast, species such as *A. cremastogyne* and *A. mandshurica*, which are expected to expand their potential ranges, should be closely monitored to assess their impacts on recipient ecosystems and to guide adaptive management responses as new habitats are colonized. At the regional scale, proactive strategies such as maintaining and restoring habitat connectivity, especially in southern and southwestern China where range contractions are most severe, can facilitate species migration and enhance resilience to environmental changes. Integrating species distribution modeling results with long-term forest management plans will provide a scientific basis for prioritizing conservation actions and adapting forest management practices to future environmental conditions. Such integrative approaches are essential for maintaining the ecological functions and genetic diversity of *Alnus* species in a changing climate [49,50,51].

### 3.4. Limitations and Future Research Directions

Despite demonstrating robust predictive capabilities, this study has certain limitations. Our research focused on five representative *Alnus* species within China, potentially limiting broader generalizations to the entire genus or global scale. Additionally, predictive accuracy may be influenced by limited occurrence data for certain species, highlighting the need for broader geographic sampling. Future research should expand species coverage and include additional ecological factors, including biotic interactions and edaphic (soil-related) variables, to further enhance predictive accuracy and ecological realism.

## 4. Materials and Methods

### 4.1. Species Distribution Data

We selected five representative *Alnus* species in China (*A. cremastogyne*, *A. ferdinandi-coburgii*, *A. mandshurica*, *A. trabeculosa*, and *A. hirsuta*) based on their ecological importance, geographic representativeness, and the availability of sufficient occurrence records for robust modeling. All distribution data used in this study were downloaded from the Global Biodiversity Information Facility (GBIF, https://www.gbif.org/; accessed 12 February 2025). Spatial autocorrelation was minimized using a thinning approach with the R package “spThin” v.0.1.0, applying a 5 km × 5 km grid resolution to ensure only one occurrence record per grid [52]. After spatial thinning, the following occurrence points were used for modeling: 91 for *A. cremastogyne*, 32 for *A. ferdinandi-coburgii*, 21 for *A. mandshurica*, 97 for *A. trabeculosa*, and 69 for *A. hirsuta* (Table 1; Figure 1). ArcGIS 10.8 (Esri, Redlands, CA, USA) was then used to generate maps of the species occurrences (Figure 1).

### 4.2. Environmental Variables

We downloaded 19 bioclimatic variables at a 2.5 min resolution for the period of 1970–2000 as current environmental variables from the WorldClim 2.1 dataset (https://worldclim.org/) (Table S1) [53]. These variables include temperature and precipitation metrics commonly used in ecological modeling. For example, temperature seasonality (bio04), a key variable in our analysis, is defined as the standard deviation of monthly mean temperatures multiplied by 100. For future climate scenarios, we used the medium-resolution Beijing Climate Center Climate System Model (BCC-CSM2-MR) from WorldClim 2.1, which provides projections under four Shared Socioeconomic Pathways (SSPs): SSP1-2.6 (Sustainable Development), SSP2-4.5 (Moderate Development), SSP3-7.0 (Partial Development), and SSP5-8.5 (General Development). We selected environmental variables for the 2090s (2080–2100) under these four scenarios, also with a resolution of 2.5 min.

### 4.3. Key Environmental Variable Selection

High multicollinearity among environmental factors may lead to model overfitting, which can undermine the predictive accuracy of statistical models [54]. Therefore, we screened and selected the main environmental variables for each species based on the initial contribution of the environmental variables in the model outputs, where percentage contributions were calculated relative to the full set of input variables, with the contributions summing to 100% (Table S2). Pearson correlation analyses (∣r∣ ≤ 0.8) were performed using the R package “corrplot”. Subsequently, variance inflation factors (VIF ≤ 5) were calculated for the key variable groups of each species using the R package “MASS” [55] to assess multicollinearity within these groups (Figure S1). Finally, we selected four environmental variables for each species for subsequent modeling (Table 2).

### 4.4. Model Parameter Optimization

The MaxEnt model is built using key parameters, including the Regularization Multiplier (RM) and Feature Combination (FC). In this study, we set the RM values from 1 to 4 and employed four FCs: LQ, LQH, LQHP, and LQHPT, yielding a total of 16 distinct parameter combinations. The feature types (L, Q, H, T, P) represent different transformations of environmental variables in MaxEnt and help control model complexity. We utilized the R package “ENMeval” [56] to assess the complexity of these 16 parameter combinations by computing indices such as the difference in Delta Akaike Information Criterion corrected (Delta.AICc), Area Under the Curve Difference (AUC.DIFF), and 10% Training Omission Rate (OR10) (Figure S2). Based on these evaluations, we selected the optimal parameter combinations for each species (Table 1).

### 4.5. Model Construction of MaxEnt

Species occurrence data along with their corresponding key environmental variables were imported into MaxEnt v.3.4.4 [57]. For each species, the optimal feature combination and regularization multiplier were determined (Table 1). We randomly selected 75% of the occurrence records for training and 25% for testing, with 20 repetitions and 5000 iterations. The results were averaged across the repetitions. In the “Projection layers directory/file” section, we chose the folder containing the environmental variables for future climate projections to construct the ecological niche models for future climate scenarios.

### 4.6. Model Accuracy Evaluation

We assessed the accuracy of the model using two methods: the area under the curve (AUC) of the receiver operating characteristic (ROC) curve and the true skill statistic (TSS) [58,59]. The AUC values ranged from 0 to 1, with higher values indicating better model performance. Specifically, an AUC between 0.5 and 0.6 suggests ineffective prediction, 0.6–0.7 indicates poor prediction, 0.7–0.8 reflects moderate prediction, 0.8–0.9 denotes good prediction, and 0.9–1 represents excellent prediction; in particular, AUC values exceeding 0.9 reflect highly accurate predictions [60,61]. TSS evaluates the overall predictive ability of the model by comparing the true positive and false positive rates. The TSS ranges from -1 to +1, with values closer to 1 indicating higher accuracy. A TSS greater than 0.75 is considered excellent, values between 0.75 and 0.40 indicate a good model, and a TSS below 0.40 suggests an ineffective model [59,62].

### 4.7. Model Output and Data Analysis

The average results from 20 replicate simulations of the MaxEnt model were imported into ArcGIS 10.8 and converted from ASC format into raster format. The suitability levels were reclassified into four categories using the reclassification tool: areas with *p*-values of 0–0.1 were classified as non-suitable; 0.1–0.3 as low suitability; 0.3–0.5 as moderate suitability; and 0.5–1 as high suitability. The distribution area of each species at each suitability level was calculated, and corresponding suitability distribution maps were generated. Cells with probability values (*p*) < 0.1 were assigned a value of 0, while those with probability values (*p*) ≥ 0.1 were assigned a value of 1. This approach enabled the construction of a presence/absence (0,1) matrix for both current and future climate scenarios, facilitating analyses of distribution area contractions and expansions for each species under varying future conditions. Finally, maps were generated to visualize the predicted expansions and contractions of suitable distribution areas from the present to future climatic conditions.

## 5. Conclusions

This study employed the MaxEnt model to predict the potential suitable distributions of five *Alnus* species under both current and future climate scenarios. Our findings emphasize temperature seasonality (bio04) as the most critical determinant shaping the potential distributions of *Alnus* species under future climate scenarios. As climate warming intensifies, significant shifts toward higher altitudes and latitudes are anticipated, though species responses vary distinctly. Specifically, *A. cremastogyne*, *A. mandshurica*, and *A. hirsuta* are projected to experience substantial range expansions, while *A. ferdinandi-coburgii* is expected to undergo notable potential habitat contraction. Effective conservation strategies and adaptive management practices must be regionally tailored to address these shifts, particularly for vulnerable species facing substantial habitat contraction. This research contributes valuable insights for conservation planning and sustainable management of forest ecosystems under climate uncertainty. Given the cosmopolitan distribution of *Alnus* species, the findings may support similar efforts in other temperate zones globally.

## Figures and Tables

**Table 1:** Distribution data, model parameters, and model accuracy estimates for *Alnus* species.

Species	Distribution Points	Parameter		AUC (SD)	TSS (SD)
		FC	RM		
*A* *. cremastogyne*	91	LQH	2	0.9457 (0.012)	0.9366 (0.026)
*A* *. ferdinandi-coburgii*	32	LQHPT	1	0.9864 (0.006)	0.9614 (0.056)
*A* *. hirsuta*	69	LQHP	1	0.9408 (0.013)	0.9408 (0.013)
*A* *. mandshurica*	21	LQ	1	0.9301 (0.032)	0.9101 (0.083)
*A* *. trabeculosa*	97	LQHPT	2	0.9629 (0.007)	0.9525 (0.032)

Note: SD, Standard deviation; FC, feature combination; RM, regularization multiplier. Feature combinations used in the model include L (linear), Q (quadratic), H (hinge), T (threshold), and P (product).

**Table 2:** Contributions and ranges of values of main environmental variables for *Alnus* species.

Species	Variable	Percent Contribution	Logistic > 0.5	Logistic Max
*A* *. cremastogyne*	bio12	39.9	806.14–1359.74	944.54
	bio06	29.3	−1.72–5.01	3.52
	**bio04**	19.3	597.87–764.80	697.69
	bio19	11.5	21.46–101.50	44.08
*A* *. ferdinandi-coburgii*	**bio04**	44	429.76–552.32	463.02
	bio06	30.8	1.43–3.95	1.26
	bio12	15.5	793.11–1091.96	878.50
	bio19	9.7	23.40–60.66	36.30
*A* *. hirsuta*	**bio04**	39.9	1244.77–1935.41	1418.34
	bio12	38.1	545.05–758.94	616.35
	bio03	16	14.80–24.84	14.80
	bio15	6	91.16–109.68	96.61
*A* *. mandshurica*	**bio04**	55.3	1428.43–1922.06	1777.50
	bio13	33.1	111.62–199.50	124.99
	bio01	7.9	−4.22–4.74	0.26
	bio03	3.7	20.58–26.84	25.7
*A* *. trabeculosa*	bio17	89	131.02–297.01	155.65
	**bio04**	5.7	710.50–890.41	820.94
	bio13	4	218.85–313.89	265.5
	bio02	1.3	7.77–9.12	8.66

Note: Bold values show key environmental variables common to all five species. Logistic > 0.5 indicates the range of the variable where the predicted suitability exceeds 0.5. Logistic max represents the value at which the model predicts the maximum habitat suitability for each variable.

**Table 3:** Suitable distribution areas for *Alnus* species under current and future climate scenarios.

Species	Scenario	Area (×10^4^ km^2^)				
		High Suitable	Medium Suitable	Low Suitable	total Suitable	Unsuitable
*A. cremastogyne*	current	32.82	40.41	102.39	175.62	788.39
	SSP1-2.6	35.59 (+8.44%)	45.03 (+11.43%)	129.30 (+26.28%)	209.92 (+19.53%)	754.10 (−4.35%)
	SSP2-4.5	39.44 (+20.17%)	52.49 (+29.89%)	149.59 (+46.10%)	241.53 (+37.53%)	722.48 (−8.36%)
	SSP3-7.0	36.38 (+10.85%)	49.89 (+23.46%)	112.82 (+10.19%)	199.09 (+13.36%)	764.92 (−2.98%)
	SSP5-8.5	39.73 (+21.05%)	51.06 (+26.35%)	102.32 (−0.07%)	193.11 (+9.96%)	770.90 (−2.22%)
*A. ferdinandi-coburgii*	current	12.15	14.05	29.90	56.10	907.91
	SSP1-2.6	0.12 (−99.01%)	5.45 (−61.21%)	13.75 (−54.01%)	19.32 (−65.56%)	944.69 (+4.05%)
	SSP2-4.5	0.28 (−97.70%)	8.17 (−41.85%)	17.62 (−41.07%)	26.07 (−53.53%)	937.94 (+3.31%)
	SSP3-7.0	0.98 (−91.93%)	4.73 (−66.33%)	17.91 (−40.10%)	23.63 (−57.88%)	940.39 (+3.58%)
	SSP5-8.5	1.14 (−90.62%)	3.90 (−72.24%)	20.96 (−29.90%)	25.99 (−53.67%)	938.02 (+3.32%)
*A. hirsuta*	current	51.32	29.38	104.60	185.29	778.72
	SSP1-2.6	69.24 (+34.92%)	60.51 (+105.96%)	75.71 (−27.62%)	205.46 (+10.89%)	758.55 (−2.59%)
	SSP2-4.5	54.86 (+6.90%)	57.23 (+94.79%)	64.26 (−38.57%)	176.36 (−4.82%)	787.65 (+1.15%)
	SSP3-7.0	66.74 (+30.05%)	65.83 (+124.06%)	94.69 (−9.47%)	227.26 (+22.65%)	736.75 (−5.39%)
	SSP5-8.5	68.93 (+34.31%)	72.78 (+147.72%)	90.93 (−13.07%)	232.64 (+25.55%)	731.37 (−6.08%)
*A. mandshurica*	current	54.93	78.72	75.73	209.37	754.64
	SSP1-2.6	116.73 (+112.51%)	52.76 (−32.98%)	117.92 (+55.71%)	287.41 (+37.27%)	676.60 (−10.34%)
	SSP2-4.5	85.58 (+55.80%)	52.06 (−33.87%)	127.93 (+68.93%)	265.57 (+26.84%)	698.44 (−7.45%)
	SSP3-7.0	59.29 (+7.94%)	44.65 (−43.28%)	156.82 (+107.08%)	260.77 (+24.55%)	703.25 (−6.81%)
	SSP5-8.5	68.65 (+24.98%)	45.64 (−42.02%)	140.13 (+85.04%)	254.42 (+21.52%)	709.59 (−5.97%)
*A. trabeculosa*	current	38.28	41.12	33.97	113.37	850.64
	SSP1-2.6	45.47 (+18.78%)	36.16 (−12.06%)	38.01 (+11.89%)	119.64 (+5.53%)	844.37 (−0.74%)
	SSP2-4.5	53.60 (+40.02%)	39.22 (−4.62%)	37.41 (+10.13%)	130.23 (+14.87%)	833.78 (−1.98%)
	SSP3-7.0	69.76 (+82.24%)	38.05 (−7.47%)	30.84 (−9.21%)	138.65 (+22.30%)	825.37 (−2.97%)
	SSP5-8.5	50.62 (+32.24%)	42.49 (+3.33%)	40.84 (+20.22%)	133.94 (+18.14%)	830.07 (−2.42%)

Note: Values in parentheses indicate the percentage change relative to the current distribution for each species under future climate scenarios.

**Table 4:** Changes in the suitable area of distribution of *Alnus* species under different future scenarios.

Species	Scenario	Area (×10^4^ km^2^)		
		Stability	Contraction	Expansion
*A* *. cremastogyne*	Current-SSP1-2.6	159.22	16.29	50.62
	Current-SSP2-4.5	156.90	18.63	84.71
	Current-SSP3-7.0	145.97	29.65	53.12
	Current-SSP5-8.5	135.18	40.44	57.95
*A* *. ferdinandi-coburgii*	Current-SSP1-2.6	19.06	37.11	0.27
	Current-SSP2-4.5	23.24	32.94	2.82
	Current-SSP3-7.0	17.75	38.42	5.885
	Current-SSP5-8.5	18.21	37.96	7.762
*A* *. hirsuta*	Current-SSP1-2.6	157.77	27.52	47.65
	Current-SSP2-4.5	146.51	38.78	29.80
	Current-SSP3-7.0	165.46	19.83	61.88
	Current-SSP5-8.5	161.43	23.86	71.15
*A* *. mandshurica*	Current-SSP1-2.6	208.66	0.33	78.15
	Current-SSP2-4.5	198.81	10.17	66.32
	Current-SSP3-7.0	191.64	17.32	68.59
	Current-SSP5-8.5	198.19	10.79	55.69
*A* *. trabeculosa*	Current-SSP1-2.6	97.85	15.51	21.78
	Current-SSP2-4.5	108.19	5.17	22.03
	Current-SSP3-7.0	111.39	1.98	27.25
	Current-SSP5-8.5	111.16	2.21	22.78

## Data Availability

All data used in this work are contained within the article and Supplementary Materials.

## Supplementary Materials

The following supporting information can be downloaded at: https://www.mdpi.com/article/10.3390/plants14111597/s1, Figure S1: Results of screening for key environmental variables for *Alnus* species; Figure S2: Optimization results of MaxEnt model parameters for *Alnus* species; Table S1: Names and definition of bioclimatic variables used in this study; Table S2: Contribution of initial environmental variables for *Alnus* species.

## Abbreviations

The following abbreviations are used in this manuscript:

AICc	Akaike Information Criterion corrected
AUC	Area Under the Curve
FC	Feature Combination
OR10	10% Training Omission Rate
RM	Regularization Multiplier
ROC	Receiver Operating Characteristic
SDM	Species Distribution Model
SSP	Shared Socioeconomic Pathway
TSS	True Skill Statistic
VIF	Variance Inflation Factor
