# Supplementary material for: Predicting Range Shifts of Five Alnus (Betulaceae) Species in China Under Future Climate Scenarios
Source: Plants (Basel). 2025 May 24;14(11):1597. doi: 10.3390/plants14111597 (PMC12157331; doi:10.3390/plants14111597)
Supplement: Supplementary file 1 [file plants-14-01597-s001.zip › plants-3607942-supplementary.pdf]

Supplementary Materials

Predicting Range Shifts of Five *Alnus* (Betulaceae) Species in China Under Future Climate Scenarios

Wenjie Yang[1,2,†], Zhilong Huang[1,†], Chenlong Fu[1], Zhuang Zhao[1], Xiaoyue Yang[1], Quanjun Hu[2] and Zefu Wang[1,*]

[1] State Key Laboratory of Tree Genetics and Breeding, Co-Innovation Center for Sustainable Forestry in Southern China, College of Ecology and Environment, Nanjing Forestry University, Nanjing 210037, China; ywj.chuck@gmail.com (W.Y.); huang20010224@gmail.com (Z.H.); chenlongfu1219@njfu.edu.cn (C.F.); njzhaozhuang@njfu.edu.cn (Z.Z.); yangxiaoyue@njfu.edu.cn (X.Y.)

[2] Key Laboratory of Bio-Resource and Eco-Environment of Ministry of Education, College of Life Sciences, Sichuan University, Chengdu 610065, China; huquanjun@scu.edu.cn (Q.H.)

* Correspondence: Zefu Wang (wangzefu@njfu.edu.cn)

† These authors contributed equally.

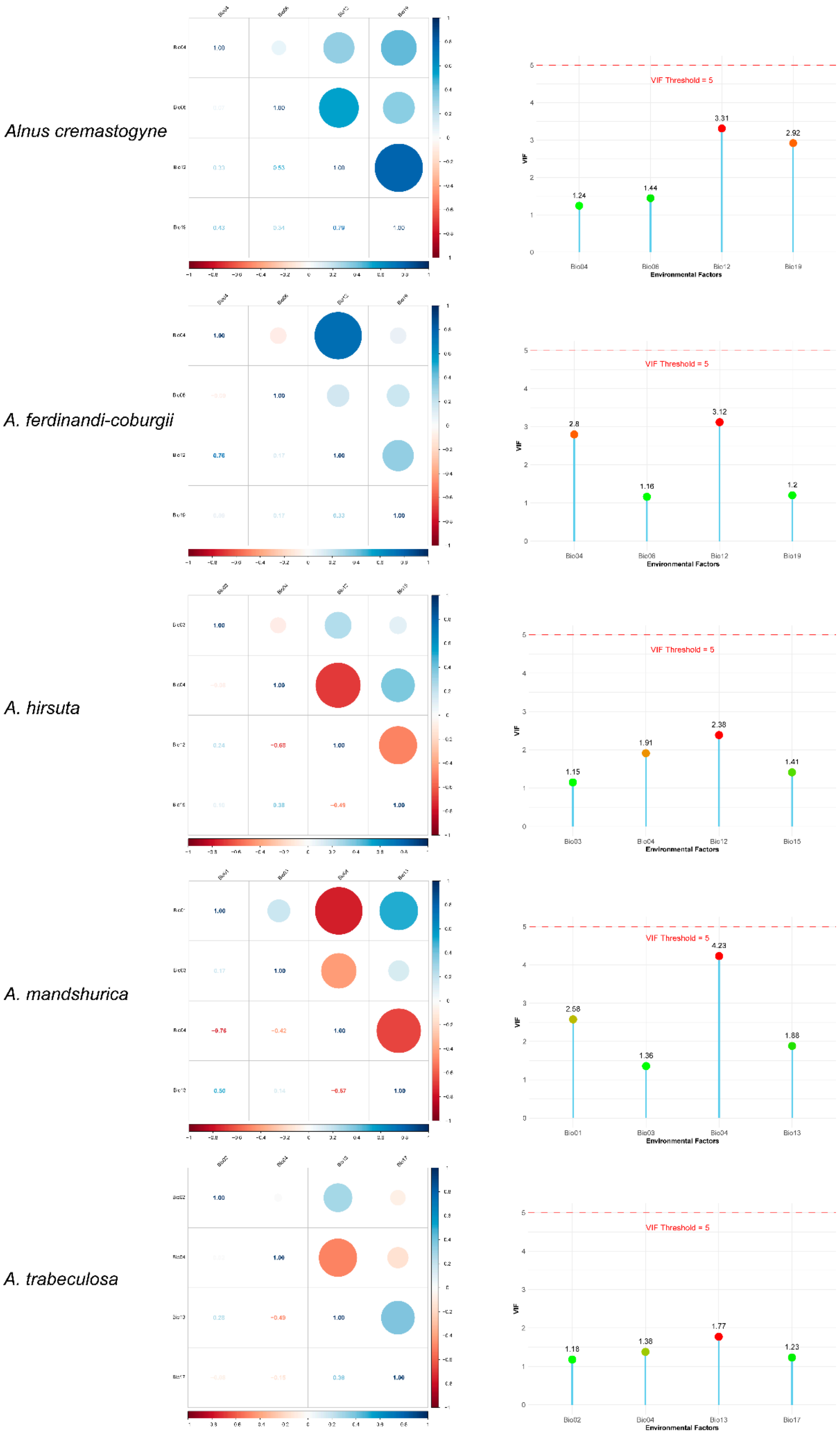

Figure S1. Results of screening for key environmental variables for *Alnus* species.

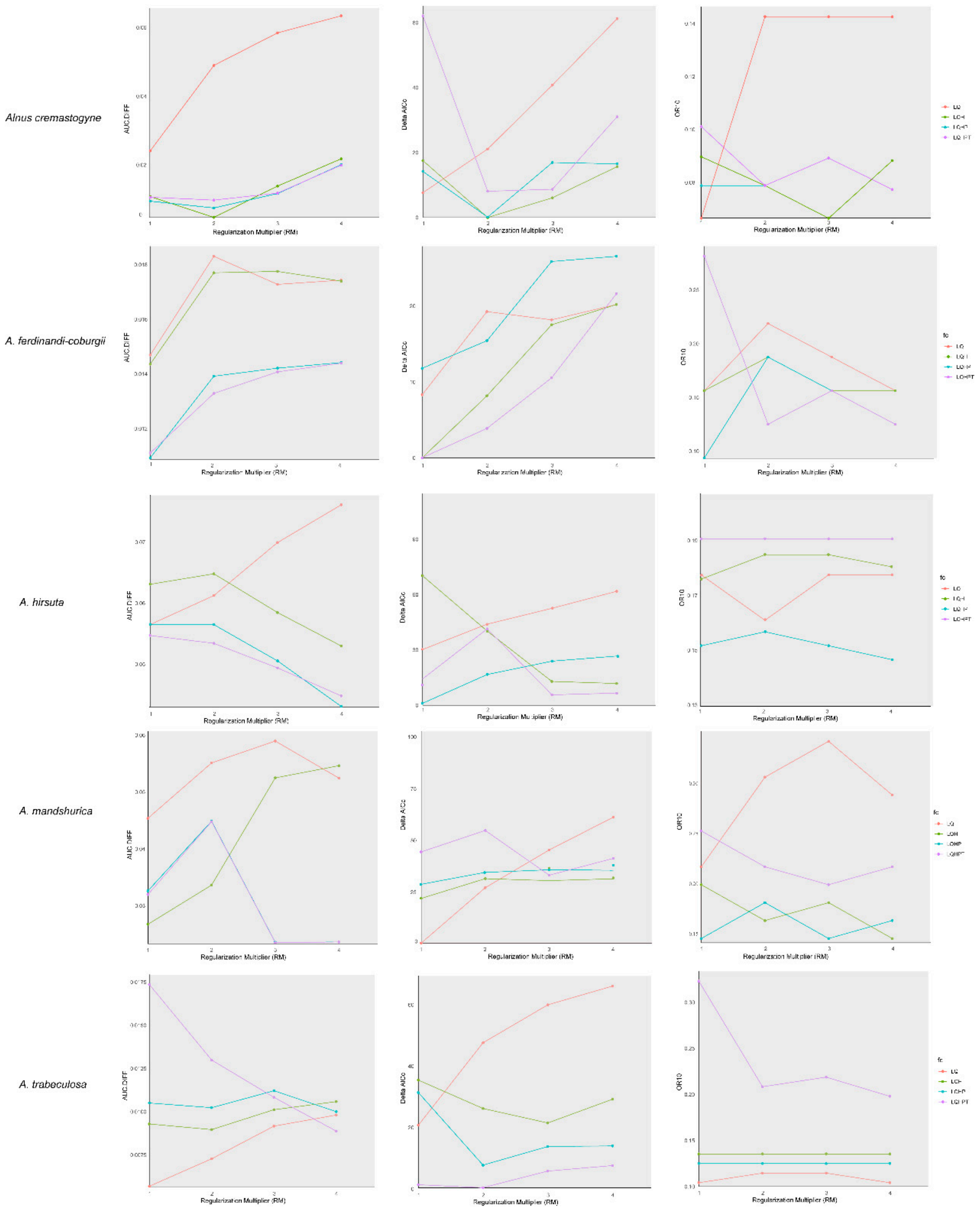

Figure S2. Optimization results of MaxEnt model parameters for *Alnus* species.

Table S1. Names and definition of bioclimatic variables used in this study.

Variables	Definition
bio1	Annual Mean Temperature
bio2	Mean Diurnal Range (Mean of monthly (max temp - min temp))
bio3	isothermality (bio2/bio7) (* 100)
bio4	Temperature Seasonality (standard deviation *100)
bio5	Max Temperature of Warmest Month
bio6	Min Temperature of Coldest Month
bio7	Temperature Annual Range (bio5-bio6)
bio8	Mean Temperature of Wettest Quarter
bio9	Mean Temperature of Driest Quarter
bio10	Mean Temperature of Warmest Quarter
bio11	Mean Temperature of Coldest Quarter
bio12	Annual Precipitation
bio13	Precipitation of Wettest Month
bio14	Precipitation of Driest Month
bio15	Precipitation Seasonality (Coefficient of Variation)
bio16	Precipitation of Wettest Quarter
bio17	Precipitation of Driest Quarter
bio18	Precipitation of Warmest Quarter
bio19	Precipitation of Coldest Quarter

Table S2. Contribution of initial environmental variables for *Alnus* species.

A. cremastogyne		*A. ferdinandi-coburgii*		*A. hirsuta*		*A. mandshurica*		*A. trabeculosa*	
**Variable**	**Contribution**	**Variable**	**Contribution**	**Variable**	**Contribution**	**Variable**	**Contribution**	**Variable**	**Contribution**
bio12	37.5	bio06	31.5	bio04	28.9	bio04	52.2	bio17	83.4
bio06	25.1	bio04	28.5	bio03	19.7	bio13	23.4	bio04	3.5
bio07	15.9	bio12	14.3	bio18	18.6	bio01	7.7	bio07	3.3
bio04	8.3	bio03	12	bio19	9.8	bio07	4.7	bio02	3.3
bio19	6.5	bio07	5.2	bio15	6.9	bio17	4	bio06	1.9
bio15	2.8	bio19	4.1	bio12	6.8	bio03	3.6	bio19	1.8
bio14	1.2	bio10	2.1	bio09	3.2	bio18	3.1	bio13	1
bio09	1	bio14	0.8	bio10	2.1	bio14	1.2	bio15	0.8
bio05	0.5	bio11	0.5	bio02	1.8	bio02	0	bio05	0.5
bio08	0.4	bio08	0.5	bio01	1.3	bio15	0	bio03	0.3
bio02	0.2	bio02	0.3	bio13	0.5	bio19	0	bio12	0.1
bio03	0.2	bio01	0	bio06	0.2	bio16	0	bio09	0
bio18	0.1	bio13	0	bio07	0.1	bio12	0	bio08	0
bio16	0.1	bio16	0	bio11	0	bio11	0	bio11	0
bio13	0.1	bio05	0	bio14	0	bio10	0	bio18	0
bio01	0.1	bio17	0	bio05	0	bio09	0	bio16	0
bio17	0	bio18	0	bio16	0	bio08	0	bio14	0
bio11	0	bio09	0	bio17	0	bio06	0	bio10	0
bio10	0	bio15	0	bio08	0	bio05	0	bio01	0
